# A bioluminescent deep-sea polychaete within the genus *Aricidea* (Paraonidae) from Minamidaito Island, Japan

**DOI:** 10.1038/s41598-025-20544-2

**Published:** 2025-10-21

**Authors:** Manabu Bessho-Uehara, Naoto Jimi, Yoshihiro Fujiwara

**Affiliations:** 1 The Frontier Research Institute for Interdisciplinary Sciences, Tohoku University, 6-3 Aramaki, Aoba, Sendai, Miyagi 980-8578 Japan; 2 Graduate School of Life Sciences, Tohoku University, 6-3 Aramaki, Aoba, Sendai, Miyagi 980-8578 Japan; 3 Sugashima Marine Biological Laboratory, Graduate School of Science, Nagoya University, 429-63 Sugashima, Toba, Mie 517-0004 Japan; 4 Centre for Marine and Coastal Studies, Universiti Sains Malaysia (USM), 11800 Gelugor, Penang Malaysia; 5 Research Institute for Global Change (RIGC), Japan Agency for Marine-Earth Science and Technology (JAMSTEC), Yokosuka, Kanagawa 237-0061 Japan

**Keywords:** Bioluminescence, Annelida, Polychaeta, Deep sea, D-ARK, Biodiversity, Zoology, Biooceanography

## Abstract

The phylum Annelida encompasses a diverse group of animals, with bioluminescent species documented in 14 families. Despite this diversity and the scattered distribution of bioluminescent lineages, little is known about the molecular biology, chemistry, morphology, ecology, and evolution of bioluminescence in annelids. During a deep-sea exploration off Minamidaito Island in the western Pacific Ocean, we discovered that *Aricidea* sp. emits green light when stimulated. The specimens were identified with the limited key morphology as a species in the genus *Aricidea*. A molecular phylogenetic analysis suggests that the specimen belongs to the *Aricidea*/*Paraonis* clade but was not nested in the described species, of which sequences were publicly available. This study is the first to report bioluminescence within the family Paraonidae.

## Introduction

Bioluminescence, the phenomenon of visible light production in living organisms, results from specific chemical reactions^1^. This ability has independently evolved over 100 times across various lineages from bacteria to chordates^2,3^, implying its adaptive fitness. In fact, luminous organisms are abundant in the ocean. High-definition cameras mounted on underwater robots have revealed that three-quarters of pelagic organisms and 30–40% of benthic individuals exhibit bioluminescence^4,5^. Recent studies highlight the potential for even greater diversity of luminous animals in the ocean, particularly within deep-sea fauna^3,6^.

The phylum Annelida, comprising approximately 20,000 described species, occupies habitats ranging from terrestrial environments to ocean depths of 10,000 m^7^. More than 100 luminous annelid species have been identified from 14 families, of which one is a sipunculid, five are clitellates, and the rest are polychaetes^8,9^. Various luminous species show diverse behaviors with bioluminescence. In addition, there are less detailed and perhaps dubious reports of bioluminescent annelids in the literature (e.g. *Alciopina* and *Rhynchonerrella* from Phyllodocidae; *Onuphis* from Onuphidae; *Aglaophamus* from Nephtyidae) because they are based on single observations in which the actual source of the light was uncertain; these reports require confirmation through renewed observation^8^. The continued accumulation of rigorously documented cases is essential for a comprehensive understanding of annelid bioluminescence.

The scattered occurrence of luminous taxa across the annelid phylogeny implies multiple independent evolutionary origins of bioluminescence. To date, the chemical substrates responsible for luminescence (luciferins) have been characterized in only three families—each with a structurally distinct type: N-isovaleryl-3-aminopropanal in Acanthodrilidae; a small peptide containing oxalic acid, lysine, modified tyrosine residue, and gamma-aminobutyric acid in Enchytraeidae; and a sulfonated tricyclic thienothiochromene in Syllidae^10–13^. The commonality or uniqueness of the luciferin provides strong evidence as to whether the evolutionary origin of bioluminescence is shared or independent^2,14^. However, the biochemistry of light production in the remaining luminous lineages remains unknown, limiting our ability to reconstruct the evolutionary history of bioluminescence within Annelida^8^. Notably, reports of bioluminescence in annelids have increased even in recent years^9,15,16^, raising the possibility that there are still undiscovered luminous species among annelids, especially in environments that have not been thoroughly explored.

Paraonidae is a group of small, sub-surface deposit feeders widely distributed from shallow to deep-sea environments and often reside in mucus tubes^17^. The family comprises seven genera—*Aricidea, Cirrophorus, Levinsenia, Paradoneis, Paraonides, Paraonis* and *Sabidius*—and includes over 150 described species^17,18^*.* Species identification within Paraonidae is challenging due to the fragility of specimens, which often results in the loss of key morphological features, and the limited understanding of ontogenetic variation in diagnostic characters. Gradual transitions between body regions and subtle morphological differences further complicate accurate identification. Given these limitations, molecular phylogenetic inference is essential to support taxonomic placement, as it provides an independent and more reliable framework for resolving relationships among paraonid taxa, especially when morphological traits are incomplete or ambiguous. Although a recent molecular phylogenetic study suggested that Paraonidae is a monophyletic group closely related to Sternaspidae, it also revealed inconsistencies with traditional morphology-based classifications within Paraonidae^19^. Within the family, *Levinsenia* is supported as monophyletic, while other genera appear paraphyletic, indicating a need for taxonomic revision^19^. The ecology and biology of this group remain poorly understood, having been studied in only a few species, and no bioluminescent traits have been reported to date^17,19^.

The seafloor around Minamidaito Island is mainly composed of limestone and features steep and complex topography, making it difficult to conduct sampling using conventional deep-sea benthic collection methods such as biological dredging^20^. During the Deep-sea Archaic Refugia in Karst (D-ARK) project, we conducted a survey to investigate the local biotic communities inhabiting the distinctive karst landscape off Daito Islands, Japan. While on the D-ARK expedition, we discovered that *Aricidea* sp. (Paraonidae) exhibited green light emission. This finding constitutes the first documentation of bioluminescence in the family Paraonidae.

## Method

### Sample collection

The two specimens used in this study were collected at depths of 461 and 539 m, respectively, on the rocky ridge offshore of Minamidaito Island, Okinawa, Japan, in May 2024 by remotely operated vehicle (ROV) *KM-ROV* on the research vessel *Kaimei* during the “D-ARK” research cruise KM24-03C Leg 2. The sediment sample was collected by an Ekman barge sediment sampler. The specimens were transferred immediately after the ROV returned on board and kept in the cold room at 4 °C on the ship until further analysis. The specimen in the sediment sample was sorted and morphologically identified at the family level under the microscope on the ship. The specimens were preserved in 70% ethanol after bioluminescence observation. The ethanol-fixed specimens were morphologically investigated for key traits for identification in the laboratory.

### Bioluminescent observation

To observe bioluminescence, the specimens were kept in the chilled seawater (~ 10 °C) and stimulated mechanically using forceps or chemically by adding a few drops of 2.5 M potassium chloride. The bioluminescence was photographed with a digital camera SONY alpha 7 s III (SONY, Tokyo, JAPAN) with a 50 mm macro lens SEL50M28 (SONY).

### DNA extraction, sequencing, and phylogenetic analysis

The total genomic DNA of a specimen was extracted from the whole body using the DNeasy Blood & Tissue kit (QIAGEN, Hilden, Germany) according to the manufacturer’s instructions. Gene fragments of *COI*, *16S*, and *18S* were amplified by polymerase chain reaction (PCR) as described in Jimi et al.^21^. The *COI* and *16S* sequences were successfully obtained (Genbank Accession No. PV168495 and PV171648, respectively); however, the genetic sequence for the *18S* region could not be determined. The newly obtained DNA sequences were aligned with publicly available sequences obtained from GenBank that were used in Langeneck et al.^19^. The phylogenetic analysis was performed following Jimi et al.^21^. In brief, all sequences were aligned using MAFFT ver. 7.205 using the E-INS-i strategy^22^. Alignment-ambiguous positions were removed using trimAL with the gappyout method^23^. A phylogenetic tree was constructed using the maximum likelihood (ML) method in the RAxML-VI-HPC program with outgroups following Langerneck et al.^19,24^. The robustness of the ML tree was evaluated by 1000 bootstrap pseudo-replicates (F option).

## Results

**Fig. 1:**
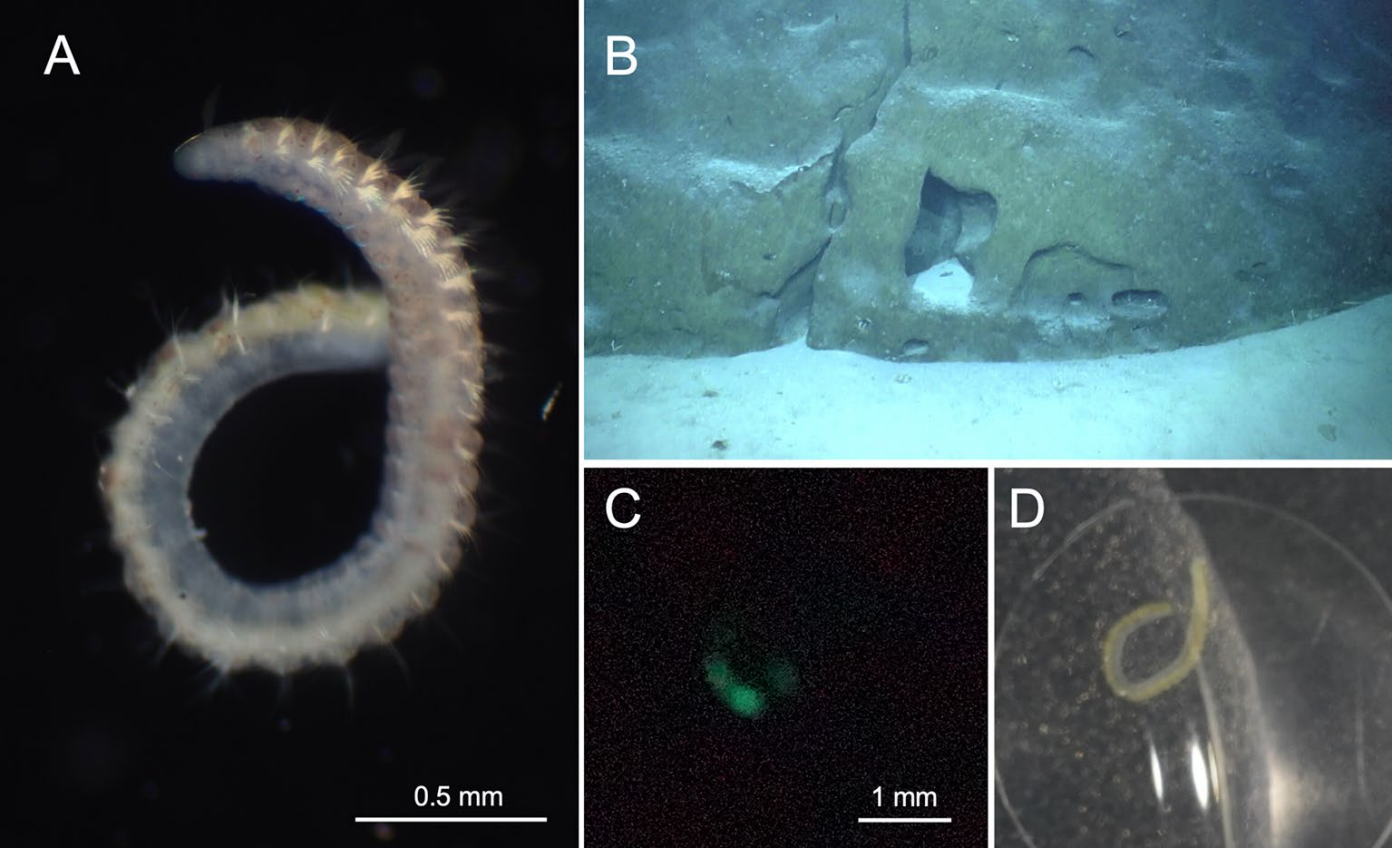
Bioluminescent benthic polychaete *Aricidea* sp. (**A**) *Aricidea* sp. photographed under bright-field illumination. (**B**) Natural habitat of *Aricidea* sp. at a depth of 461 m off Minamidaito Island (25° 57.48′ N 131° 20.60′ E). (**C**) The green bioluminescence of *Aricidea* sp. was induced by stimulating with a drop of 2.5 M KCl. Photographed using a digital camera SONY alpha 7 s III: exposure time 10 s, f/2.8, ISO 102,400. (**D**) The same frame as (**C**) under bright-field illumination.

The polychaete specimens of *Aricidea* sp. (Fig. 1A) was collected from sediment at depths of 461 m and 539 m off Minamidaito Island (Fig. 1B). The sediment consisted of coral skeleton and shell fragments, with no mud present. Since the specimens were incomplete and in poor condition, detailed morphological characteristics remain unclear. However, they possess a conical prostomium, a median antenna, three pre-branchial segments, and simple capillary chaetae. These morphological features align closely with the diagnostic traits of the genus *Aricidea*^25^. Although *Aricidea* contains several subgenera, the current condition of the specimen did not allow for identification to subgenus or species level.

**Fig. 2:**
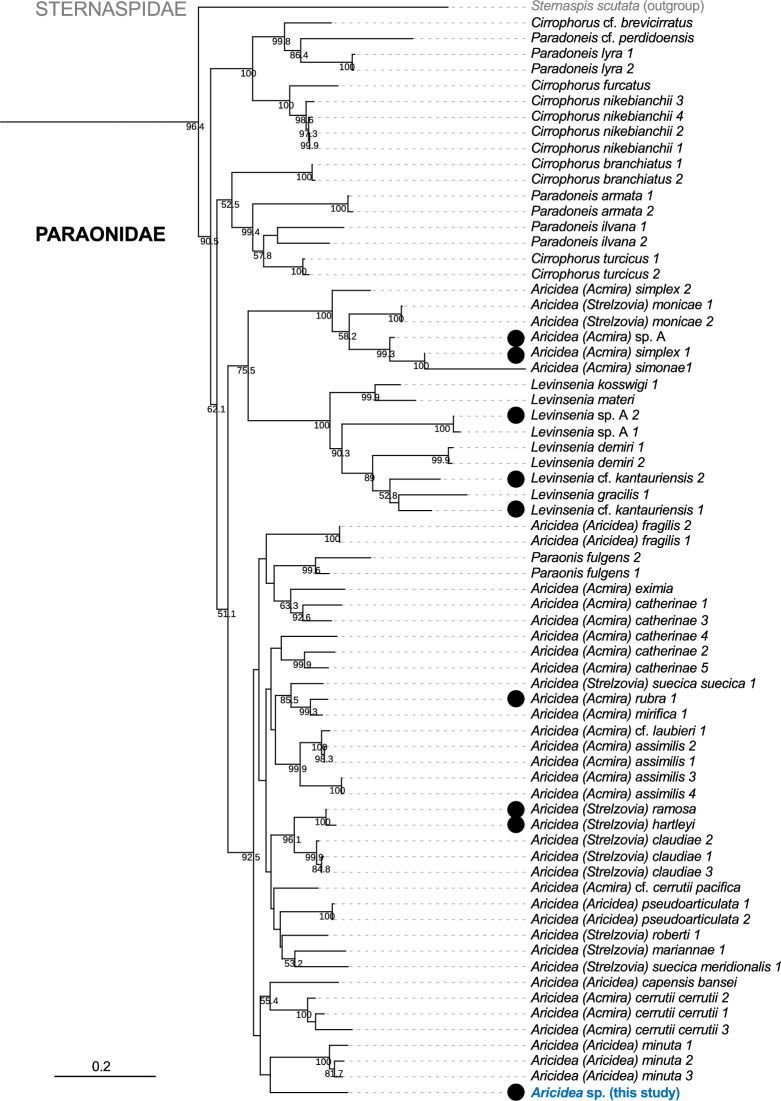
Phylogenetic tree of Paraonidae constructed using the maximum likelihood method. Bootstrap values (> 50%) are indicated at the nodes. The Genbank Accession Numbers for *Aricidea* sp. in this study are PV168495 (*COI*) and PV171648 (*16S*). The clade including Paraonidae and the most closely related taxa–Sternaspidae–was pruned. The samples collected below 200 m depth were indicated as closed black circles. All the metadata is available from this study and Langeneck et al.^19^.

In order to determine the phylogenetic position of the specimen, we performed the molecular phylogenetic analysis using *COI*, *16S*, and *18S* genes. The maximum likelihood tree with the publicly available data revealed that this specimen is nested within a clade composed of multiple *Aricidea* species (Fig. 2), supporting the morphological identification. The species in the family Paraonidae form a monophyletic group, closely related to Sternaspidae. The systematics of *Aricidea* has been debated, with suggestions of synonymy with *Paraonis*^19^. However, the issue remains unresolved^17,25^. Therefore, we refer to the specimen as *Aricidea* sp. in this study. Members of the family Paraonidae, including *Aricidea*, typically inhabit the sediment surface or its uppermost layer and often reside in mucus tubes^17^.

Bioluminescence was observed from *Aricidea* sp. upon chemical stimulation with potassium chloride (Fig. 1C–D). The whole body of the specimens emitted a dim green light for a few seconds after the stimulation. We tested two specimens independently, and both individuals emitted a dim green light. We attempted but were unable to determine the bioluminescence spectrum due to its low intensity. No specific fluorescence under ultraviolet light (excitation wavelength = 365 nm) was detected before or after the bioluminescent response, apart from chaetal autofluorescence.

## Discussion

We discovered bioluminescence in the *Aricidea* sp. for the first time in the family Paraonidae. The weak bioluminescence can be a character of this species, but it is also possible that the extreme stress on the specimens during the sample collection process (vigorously vortexed for suction sampling, drastic change in temperature, light, and pressure between deep-sea and on shipboard environments). These stressors might have triggered bioluminescence during the sample collection process, leading to the consumption of luciferin and luciferase before our testing. Ideally, bioluminescence should be observed in its natural habitat, but in situ observations of benthic animals, especially those living in sediment in the deep sea, remain challenging due to technological and financial constraints^3,26^.

Some luminous polychaetes which emit greenish light show fluorescence after bioluminescence. For example, a luminous fireworm *Odontosyllis undecimdonta* (Syllidae) produces green bioluminescent fluid that has green fluorescence^13^. A luminous scale worm *Harmothoe* spp. (Polynoidae) emits blue-green light on the scale. After bioluminescence is triggered, its scales possess blue-green fluorescence^27,28^. In contrast, *Aricidea* sp. did not show any detectable fluorescence following bioluminescence in our observations. This may suggest that the bioluminescence system in this species differs in chemical composition—such as the luciferin or associated molecules—from those in species that do exhibit fluorescence. However, alternative explanations, such as specimen degradation or physiological stress, cannot be excluded. Given that fluorescence is typically easier to detect due to the high intensity of excitation light, the absence of fluorescence despite confirmed bioluminescence is unlikely to be due to limited detection sensitivity. Nevertheless, chemical analyses are necessary to determine the nature of the bioluminescent system in *Aricidea* sp. Additionally, green autofluorescence was observed in the chaetae, as in other annelid species including non-luminous taxa, and is therefore likely unrelated to the bioluminescent mechanism.

Our molecular phylogenetic analysis suggested that *Aricidea* sp. examined in this study belongs to the family Paraonidae. To our knowledge, no prior studies have investigated bioluminescence in this group^8,19,25,29^. The phylogenetic position of the family Paraonidae within Annelida, based on molecular analyses, remains unresolved^30^. While Paraonidae is likely part of Sedentaria, its relationships within this group remain unclear. As a result, it is currently not possible to discuss the evolutionary implications of bioluminescence across Annelida comprehensively, and we therefore refrain from such discussions in this study.

The ecological role of bioluminescence in *Aricidea* sp. is unknown. The greenish bioluminescence has been reported in benthic annelids such as *Pontodrilus, Odontosyllis*, *Terrebella, Tharyx, Nephasoma*, and polynoids. Several intraspecific and interspecific roles of bioluminescence in annelids have been hypothesized but detailed observations and experimental evidence remain scarce^8^. The only documented intraspecific role of bioluminescence in luminous polychaetes is observed in *Odontosyllis* spp., where it functions as a courtship display^31^. Considering that the eyes of *Aricidea* sp. are not as well developed as those of *Odontosyllis*, it is unlikely that this species relies on bioluminescence for intraspecific communication. On the other hand, many luminous annelids and other benthic luminous animals emit light upon distracting stimulation, thus the ecological roles of the bioluminescence are considered as interspecific roles for defensive roles^8^. It is hypothesized that bioluminescence in annelids serves defensive functions such as startling predators by a flash of light, misdirecting predators by sacrificial luminous lures, or acting as an aposematic signal. The bioluminescence of the *Aricidea* sp. might also be a defensive role. However, as with other benthic bioluminescent polychaetes, many details remain unknown.

Much remains unknown about the luminescence of *Aricidea*, necessitating further observations and experiments to clarify its mechanisms, ecological roles, and evolution. However, the specimens inhabit deep-sea environments, in which sampling opportunities are limited, making detailed study challenging. Relatively minority of the members of *Aricidea* and other Paraonidae species occur in the deep environment (Fig. 2, filled black circles), whereas a majority of the members in the Paraonidae occur in shallower waters. For example, *Aricidea (Aricidea) minuta* and other *Aricidea* spp. closely related to the *Aricidea* sp. found in this study were found from shallow-water environments: from the intertidal zone down to 25 m depth^19^. It remains unclear whether any of these closely related shallow-water species are bioluminescent, but if such species are identified, they could serve as valuable model organisms for elucidating the ecological, genetic, and evolutionary underpinnings of luminescence in *Aricidea* within the *Paraonidae.*

Our study demonstrates that Paraonidae constitutes the fifteenth annelid family known to include luminous species, further highlighting the remarkable diversity of bioluminescence within Annelida. The discovery of an additional bioluminescent lineage opens new avenues of inquiry across multiple disciplines—including biochemistry, genetics, histology, morphology, and behavioral ecology—by providing a comparative framework. From the perspective of evolutionary novelty, investigations into the bioluminescence mechanisms of the species in *Aricidea* are especially promising; elucidating the molecular and cellular basis of its bioluminescence will therefore be an important priority for future research.

## Conclusion

In this study, we discovered a new bioluminescent species. The specimens of *Aricidea* sp. emitted dim green light upon chemical stimulation. The bioluminescent capability in the family Paraonidae is reported here for the first time. The genus *Aricidea* contains other species that inhabit both shallow and deep regions but have not been examined for bioluminescence, raising the possibility that bioluminescent species may also exist in shallow waters. Shallow-water bioluminescent species could serve as model species for elucidating the physiological, chemical, and ecological aspects of bioluminescence in this group. Future research should focus on the detailed taxonomic classification of this bioluminescent organism, analysis of its luminescence mechanism, and a thorough investigation of its ecological role.

## Data Availability

All data generated or analyzed during this study are included in this published article.
